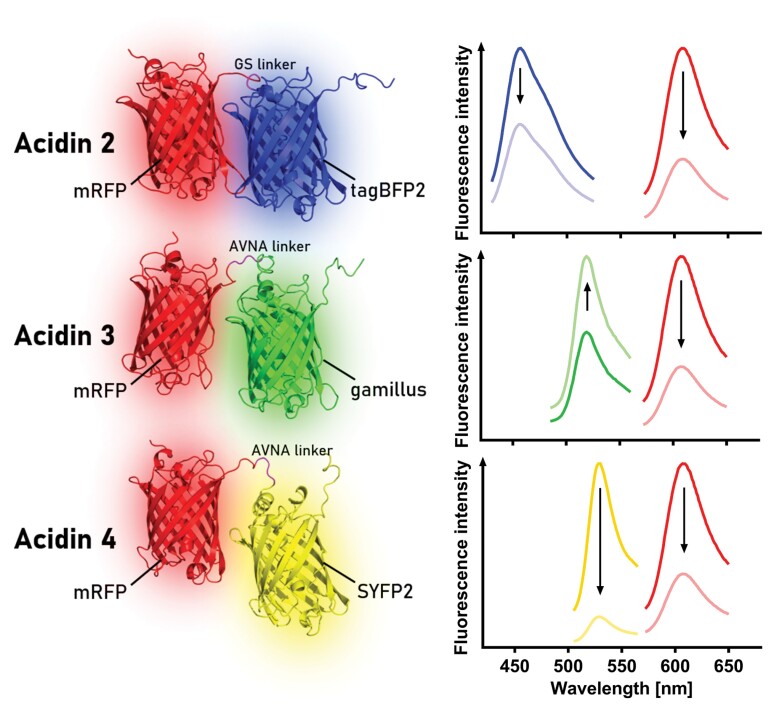# Biosensing on acid: fluorescent protein probes for low pH environments

**DOI:** 10.1093/jxb/erac409

**Published:** 2022-12-08

**Authors:** Christopher Bell, Jan Multhoff, Markus Schwarzländer

**Affiliations:** Institute of Plant Biology and Biotechnology, University of Münster, Schlossplatz 8, D-48143 Münster, Germany; Institute of Plant Biology and Biotechnology, University of Münster, Schlossplatz 8, D-48143 Münster, Germany; Institute of Plant Biology and Biotechnology, University of Münster, Schlossplatz 8, D-48143 Münster, Germany

**Keywords:** Apoplast, biosensor, fluorescence, live imaging, pH

This article comments on:

**Moreau H, Gaillard I, Paris N.** 2022. Genetically encoded fluorescent sensors adapted to acidic pH highlight subdomains within the plant cell apoplast. Journal of Experimental Botany **73,** 6744–6757. https://doi.org/10.1093/jxb/erac210

**pH determines the function of most biomolecules and life processes. In plants, growth, nutrient uptake, photosynthesis, respiration, membrane transport, and organismal interactions are all underpinned by active pH regulation. Even though pH measurements are central to plant physiology, measurements have often been limited *in planta*, where pH microenvironments are generated through the intricate cellular organization. Precise genetic targeting of fluorescent protein biosensors has provided unique resolution, but most sensors do not work for pH values below pH 5. Moreau *et al*. (2022) developed a family of low-pH biosensors, named Acidins, that expand the accessible pH range down to pH 3. The biosensors were deployed to the tobacco and Arabidopsis apoplast, revealing a structured pH landscape and giving a glimpse of the future discoveries that low-pH biosensing may hold in store.**

## pH gradients are a fundamental driver of plant cell physiology

A textbook example for the importance of cellular pH regulation is the acid growth theory of auxin. It explains turgor-mediated cell expansion through an apoplastic acidification event that allows loosening of the plant cell wall (Cleland, 1971; Hager *et al.*, 1971). Recently, low pH was reported to also be a prerequisite of auxin perception in the apoplast, which governs H^+^-ATPase activation (Friml *et al.*, 2022). More generally, pH gradients across membranes in living cells are actively generated and maintained through ATP hydrolysis by H^+^-ATPases that span the plasma membrane and the endomembranes. Most of the ATP required is generated by H^+^ gradients in the mitochondria in the first place, as well as—indirectly—the chloroplasts. Respiratory and photosynthetic electron transport drive H^+^ pumping across the inner mitochondrial membrane and the thylakoid membranes, respectively. The resulting membrane gradient fuels ATP synthesis via H^+^- driven ATP-synthases. pH has global consequences on the status of most biomolecules in cells, rendering pH a systems parameter that connects energy transformation, metabolism, membrane transport, and signal transduction. Yet, despite its ubiquitous importance, pH has remained a remarkably enigmatic parameter in cell physiology. This is not just due to the complex chemical interactions by which the free proton concentration is determined in biological systems, but also to the methodological challenge of measuring pH in living plant cells with the required spatial resolution.

## Fluorescent protein biosensing of pH—a game changer with limits

The development of genetically encoded biosensors based on fluorescent proteins has opened a new chapter for *in vivo* pH measurements. After the early realization that green fluorescent protein (GFP) can be used as an intracellular pH indicator (Kneen *et al.*, 1998; Llopis *et al.*, 1998), pHluorin was developed as a dedicated pH sensor (Miesenbock *et al.*, 1998). pHluorin (p*K*a 6.8) enables pH measurements within a range between ~5.5 and 7.5, and has been used extensively for pH analyses in several subcellular compartments of different model systems (Box 1). While pH values of the cytosol and the endoplasmic reticulum (ER) lumen were determined as mildly alkaline, the *trans*-Golgi network and early endosome were found to maintain acidic conditions (Martiniere *et al.*, 2013; Shen *et al.*, 2013). Apoplastic pH near the plasma membrane was reported to range from 6.0 to 6.4 (i.e. less acidic than in the bulk apoplastic space) in Arabidopsis mature root cells (Martiniere *et al.*, 2018). Those observations indicated a heterogenous pH landscape in the apoplast and led to the suggestion that localized pH values as low as 4 may be reached. Even though the apparent spatial heterogeneity in apoplast pH raised several new questions, the pHluorin biosensor reached its sensitivity limit at the more acidic conditions. One strategy of expanding the available repertoire of pH sensors has been to harness the fact that the signal of fluorescent proteins is quenched under acidic conditions, yet with different p*K*a values that are characteristic for a given fluorescent protein variant. The concept was introduced by linking monomeric red fluorescent protein 1 (mRFP1) and enhanced GFP (EGFP) through translational fusion to give rise to the ratiometric tandem biosensor pHusion (Gjetting *et al.*, 2012). Due to its sensitivity range between ~7.5 and 4.5, pHusion allowed pH measurements in both the cytosol and the apoplast (Gjetting *et al.*, 2012), and subsequently the endomembrane system (Luo *et al.*, 2015). More recently pH-Lemon was engineered following the tandem concept by linking mTurquoise2 and enhanced yellow fluorescent protein (EYFP), which allows pH measurements in a range from ~7 to 4, and was exploited for subcellular pH measurements of different mammalian cell lines (Burgstaller *et al.*, 2019). This sensor not only relies on the differential pH sensitivity of mTurquoise2 versus EYFP fluorescence but Förster resonance energy transfer (FRET) from mTurquoise2 to EYFP enables particularly elegant ratiometric or lifetime analysis.

Since the available pH biosensor repertoire has delivered evidence for pH gradients, microdomains, and transients in the alkaline, neutral, and mildly acidic pH range (Behera *et al.*, 2018; Elsässer *et al.*, 2020, Preprint; Monshausen *et al.*, 2011; Poburko *et al.*, 2011; Rieger *et al.*, 2014; Schwarzländer *et al.*, 2012), a similarly heterogeneous and dynamic situation is likely to apply also for the more strongly acidic cellular milieus. Even though some of the key regulation and signalling events that underpin plant growth, development, and survival are localized in acidic compartments, their exploration has been limited by the availability of sensors with a matching pH sensitivity profile.

## New tandem fluorophore biosensors for low physiological pH

Moreau *et al*. (2022) engineered a sensor family of three tandem biosensors, named Acidin2, 3, and 4, to cover the acidic pH range. All three sensors are based on an N-terminal mRFP (p*K*a 4.5), which is fused to three different fluorescent proteins of distinct colour and differential pH sensitivity (tagBFP2 p*K*a 2.7, gamillus p*K*a 3.4, and SYFP2 p*K*a 6.0) via either a Gly–Ser (GS) or an Ala–Val–Asn–Ala (AVNA) linker (Box 2). The resulting tandem biosensors have p*K*a values of 4.4, 4.5, and 5.6 to cover the acidic physiological pH range more broadly than those sensor families that had been hitherto available (Box 1). Most importantly, they extend the reach into the acidic range. Based on the *in vitro* analysis of the purified sensor proteins, the new sensor family can be used for pH measurements in a range from pH 6.5 down to pH 3.0. Since the pH ranges of the individual sensors overlap, the most suitable sensor for a specific biological phenomenon can be chosen. Towards the neutral pH range, overlap with pHluorin results in the coverage of a pH range from pH 3 to pH 8. In contrast to pH-Lemon (Burgstaller *et al.*, 2019), no major pH-dependent FRET was detected between the two fluorophores.

To put the three Acidins to the test *in planta*, Moreau *et al*. expressed the sensors transiently in tobacco leaf epidermis and stably in Arabidopsis. The proteins were targeted to the apoplast either in their free secreted form or anchored to the plasma membrane. For further analysis, a focus was set on Acidin2, which proved particularly useful in resolving the highly acidic pH conditions in the apoplast, while showing reliable localization. In contrast, Acidin3 partially mislocalized to the vacuole both in tobacco and in Arabidopsis. Exploiting the tobacco epidermal system, the free and the bound Acidin2 variants indicated a difference between the two microenvironments of ~0.6 pH units with pH 4.4 for the freely diffusing sensor that is exclusively detected in the anticlinal regions of the epidermis and pH 3.8 for the plasma membrane-anchored sensor that monitors the entire thickness of the epidermis. A steep characteristic pH gradient was further apparent when moving from the leaf surface (pH 6.0) deep into the anticlinal wall of the epidermal cell layer (pH 4.2), as indicated by the plasma membrane-anchored sensor. This gradient was dependent on H^+^-ATPase activity since it was flattened when H^+^-ATPase was hyperactivated by fusicoccin, suggesting that H^+^-ATPase activity may be differentially regulated along the anticlinal plasma membrane. Straight regions of the apoplast between the epidermal pavement cells showed a lower pH than curved regions. This difference was not abolished by fusicoccin, suggesting that parameters other than H^+^-ATPase activity, such as H^+^-ATPase distribution or the biochemical composition of the cell wall, may be causative. Those observations provide proof-of-concept that the Acidin biosensors are able to shed new light on the apoplast as a highly structured and dynamic cell physiological setting, in which important regulation and signalling events occur. To investigate the role of apoplastic pH dynamics in cell growth, primary roots of the transgenic Arabidopsis lines for Acidin2 served as a model. A characteristic apoplastic pH landscape was apparent starting from acidic in the root cap (less than pH 4), to more alkaline in the meristem (pH 5.4), to more acidic in the transition zone (pH 5.0) and the elongation zone (pH 4.6). Since the observed apoplastic pH environment was less acidic in Arabidopsis roots than in tobacco leaf epidermis, cell growth analyses were performed with Acidin4 anchored in the plasma membrane, showcasing a strength of the sensor family with different pH optima. Gravitropic responses leading to auxin-mediated changes in cell expansion were mirrored by alkalinization of the inner face of the root tip (pH 5.35), while the outer face acidified (pH 5.2), as detected by the plasma membrane-anchored Acidin4 sensor. Those observations refine previous reports based on biosensing approaches (Monshausen *et al.*, 2011; Barbez *et al.*, 2017) and showcase the power of the Acidin sensor pH family for mechanistic exploration of pH dynamics and pH-dependent functions.

## New sensor variants to explore strange new pH worlds

The tandem concept that was adopted by Moreau *et al*. (2022) provides a blueprint for future refinement. Since Acidin3 was found to mistarget to the vacuole, strategies to avoid such artefacts may include targeted mutagenesis and directed evolution approaches to remove cryptic signal sequences. Further, alternative tandem combinations may be envisaged, for example by replacing gamillus by phiLOV3 (p*K*a 3.3). A sensor in which gamillus is replaced by T-Sapphire (p*K*a 4.9) may bridge the remaining sensitivity gap between Acidin3 and 4. Fluorescent protein variants with particularly low p*K*a values, such as mBlueberry 2 (p*K*a 2.5) or mCRISPRed (p*K*a 2.1), may be interesting as building blocks for future tandem sensor designs. Importantly, each of those combinations will require dedicated *in vitro* and *in vivo* characterization, since strict rational design of biosensor properties remains a major challenge. Moreau *et al*. (2022) provide a useful guide to such characterizations. In the future, the Acidin family will enable live monitoring of pH in the various acidic subcellular environments. To be used to their full potential, the sensors deserve to be targeted also to subcellular spaces other than the apoplast, such as specific compartments of the endomembrane system or the thylakoid lumen (Box 1). Fusion with specific target proteins may allow exploration of their immediate, individual pH nanoenvironment, which may be of particular interest for membrane proteins but also protein complexes and proteins in membrane-less compartments. By contrasting the readout from freely diffusible and plasma membrane-anchored pH sensors, Moreau *et al*. (2022) have made a decisive step towards the exploration of pH gradients within a continuous subcellular space. Future analysis must reveal to what extent pH gradients between membrane and protein microenvironments are an exception, or rather the rule. Even though proton diffusion is extremely fast, stable gradients may be generated around proton pumps, transporters, or enzyme complexes that actively release or consume protons. A particularly intriguing compartment for future exploration will be the thylakoid lumen which strongly acidifies as a result of photosynthetic proton pumping (Kramer *et al.*, 1999). Yet, the specific luminal pH dynamics in individual tissues, cell types, chloroplasts, or even the different thylakoid systems within a single chloroplast remain largely uncharted, even though they are likely to hold fundamental information on how photosynthesis works under changing environments.

**Box 1. pH range covered by Acidin biosensors and acidic subcellular sites for sensor deployment**

Acidin2, 3, and 4 have particularly low p*K*a values (p*K*a 4.4, 4.5, and 5.6. Their sensitivity in low pH environments sets them apart from widely used pH biosensors, such as pHluorin (p*K*a 6.8) and expands the pH range for biosensing in acidic cell compartments. The three Acidins taken together cover a pH range from pH 6.5 down to pH 3.0, while each of the sensor variants is responsive within a range of ~2.0 pH units. The sensors enable the exploration of acidic cellular environments (indicated in red) including the apoplast (pH 4.0–6.4; Martinière *et al*., 2018), the thylakoid lumen (pH 5.2–6.1; Kramer *et al.*, 1999), the Golgi (pH 6.0–6.8; Martiniere *et al.*, 2013; Shen *et al.*, 2013), the endosome (pH 5.5–6.5; Casey *et al.*, 2010), the pre-vacuolar compartment/multivesicular bodies [pH 6.2 in Arabidopsis protoplasts from cell culture (Shen *et al.*, 2013) and pH 6.7 in tobacco epidermis (Martinière *et al*., 2013)], and the vacuole [pH 5.2–5.7 in Arabidopsis protoplasts from cell culture (Shen *et al.*, 2013) and pH 6.0 in tobacco epidermis (Martiniere *et al.*, 2013)]. Moreau *et al*. (2022) focused on pH measurements in the apoplast. The sensors were either immobilized to the plasma membrane (PM-Apo) or secreted to allow for free diffusion (Apo).

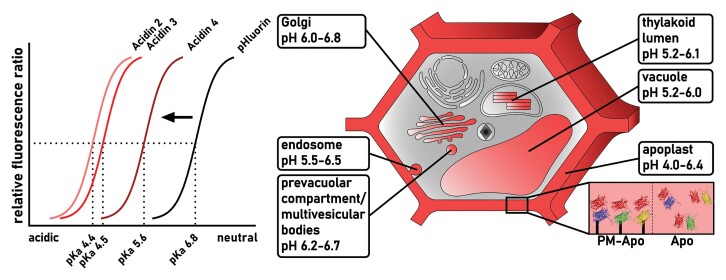

**Box 2. Acidin variants and their spectroscopic response to acidification**

Acidin2, 3, and 4 are composed of two fluorescent proteins of different pH sensitivity connected by a linker. All sensors share an N-terminal mRFP which is fused to either tagBFP2, gamillus, or SYFP2. The combination allows for ratiometric readout based on differential pH sensitivities of the two fluorophores in the acidic pH range. For Acidin2, the mRFP/tagBFP2 signal ratio is calculated, as the mRFP signal decreases more strongly than tagBFP2 when pH is lowered, leading to a ratiometric decrease in emission. Acidin3 is based on an inverse ratiometric readout as, with a drop in pH, the gamillus signal intensity increases while it decreases for mRFP. Acidin4 is based on the same principle as Acidin2; however, the SYFP2 emission intensity decreases more strongly than mRFP emission intensity. The arrows indicate the direction of the emission intensity change during acidification. Structures were predicted by Robetta (RoseTTAFold; Baek *et al.*, 2021) and visualized with PyMOL (version 2.5.2; https://pymol.org).